# The Importance of Multifrequency Impedance Sensing of Endothelial Barrier Formation Using ECIS Technology for the Generation of a Strong and Durable Paracellular Barrier

**DOI:** 10.3390/bios8030064

**Published:** 2018-07-04

**Authors:** Laverne D. Robilliard, Dan T. Kho, Rebecca H. Johnson, Akshata Anchan, Simon J. O’Carroll, Euan Scott Graham

**Affiliations:** 1 Centre for Brain Research, Faculty of Medical and Health Sciences, University of Auckland, Auckland 1023, New Zealand; l.robilliard@auckland.ac.nz (L.D.R.); d.kho@auckland.ac.nz (D.T.K.); rebecca.johnson@auckland.ac.nz (R.H.J.); a.anchan@auckland.ac.nz (A.A.); s.ocarroll@auckland.ac.nz (S.J.O.); 2 Department of Molecular Medicine and Pathology, School of Medical Sciences, Faculty of Medical and Health Sciences, University of Auckland, Auckland 1023, New Zealand; 3 Department of Anatomy and Medical Imaging, University of Auckland, Auckland 1023, New Zealand

**Keywords:** ECIS, resistance, blood brain barrier, endothelial, ZO-1, CD144

## Abstract

In this paper, we demonstrate the application of electrical cell-substrate impedance sensing (ECIS) technology for measuring differences in the formation of a strong and durable endothelial barrier model. In addition, we highlight the capacity of ECIS technology to model the parameters of the physical barrier associated with (I) the paracellular space (referred to as R_b_) and (II) the basal adhesion of the endothelial cells (α, alpha). Physiologically, both parameters are very important for the correct formation of endothelial barriers. ECIS technology is the only commercially available technology that can measure and model these parameters independently of each other, which is important in the context of ascertaining whether a change in overall barrier resistance (R) occurs because of molecular changes in the paracellular junctional molecules or changes in the basal adhesion molecules. Finally, we show that the temporal changes observed in the paracellular R_b_ can be associated with changes in specific junctional proteins (CD144, ZO-1, and catenins), which have major roles in governing the overall strength of the junctional communication between neighbouring endothelial cells.

## 1. Introduction

An important area of endothelial biology is understanding how the physical properties of an endothelial barrier are formed and regulated under normal physiological homeostasis as well as during disease states, acute infections, and by a range of other insults (e.g., drugs, physical trauma) [1]. For in vitro-based research, this requires advanced technologies to investigate the barrier function, ideally in real-time and in a noninvasive continuous manner. In this paper, we highlight the power and utility of electrical cell-substrate impedance sensing (ECIS) biosensor technology to reveal massive differences in the capability of brain endothelial cells to form a barrier by subtly varying the ingredients of the culture medium.

ECIS biosensor technology was developed by Applied Biophysics (USA) and has a broad range of applications depending on the cell type of interest. For endothelial cells and epithelial cells, ECIS technology is capable of measuring and modelling a number of barrier-related parameters as well as changes in cellular behaviour [2]. This is possible because ECIS is a multifrequency AC impedance biosensor. Impedance (Z; ohms) is comprised of resistance (R) and capacitance (C). Capacitance provides measurements related to the overall coverage of the well by the cell layer, whereas resistance is indicative of the barrier functions of the endothelial cells. Due to the multifrequency nature of ECIS, the impedance data can be mathematically modelled to indicate the contribution of the paracellular junctional space, the basal adhesion of the cells, and the cell membrane to the total impedance to current. These modelled values are referred to as R_b_, α, and C_m_, respectively [3]. Other impedance biosensors such as the xCELLigence RTCA system from ACEA, which we have used extensively for a variety of research applications, are not capable of conducting multifrequency impedance measurements and therefore cannot derive the contributions from the various cellular barrier compartments [4,5,6,7,8]. ECIS biosensor technology is the only commercially available system capable of measuring and modelling these parameters, and in this paper, we highlight a simple example of its power to do so. 

Brain endothelial cells, like all endothelial cells, have specific intrinsic properties that control the formation and function of the barrier between the brain parenchyma and the blood [1,9]. In order for these properties to be realised, brain endothelial cells must be cultured under suitable culture conditions to produce a strong and durable barrier [10,11]. Here, we demonstrate the power of ECIS biosensor technology to reveal that subtle differences in culture conditions have substantial effects on barrier formation of an immortalised brain endothelial cell line, which we have extensively characterised previously [6,7,8,12]. Importantly, the R_b_ component of the barrier was the parameter affected greatest by the media modifications, and here, we show how rapidly these changes can occur. Furthermore, these differences translate into changes in expression of key paracellular junctional proteins which were assessed using immunocytochemistry. This paper exemplifies the necessity of ECIS measurements being able to separate changes occurring at the paracellular vs. basal adhesion level, the value of the measurements being conducted in real-time to provide a temporal profile of the barrier formation, and correlating the subsequent changes that occur to overall impedance measurements. 

## 2. Materials and Methods

### 2.1. Cell Culture

Human cerebral microvascular endothelial cells (HCMVEC’s) (cat# T0259, ABM Good, Richmond, BC, Canada) were grown and passaged in T75 flasks coated with 1 µg/mL rat tail collagen I (Gibco, Waltham, MA, USA). Cells were maintained in M199 media (Gibco, Waltham, MA, USA) supplemented with 10% FBS, 1 µg/mL hydrocortisone (Sigma, St Louis, MO, USA), 80 µM butyryl cAMP (Sigma, St Louis, MO, USA), 10 µg/mL heparin (Sigma), 1 ng/mL human EGF (Peprotech, Rocky Hill, NJ, USA), 3 ng/mL human FGF (Peprotech, Rocky Hill, NJ, USA), and 2 mM Glutamax (Gibco, Waltham, MA, USA). This media is referred to as Enriched Media. In some experiments, different media were used as described in the figure legends. Minimal Media contained M199 media supplemented with 2% FBS, 39 ng/mL hydrocortisone, and 80 µM butyrul cAMP. Cells were maintained at 37 °C in a humidified 5% CO_2_ incubator.

### 2.2. ECIS Theory and Modelling

**Figure 1:**
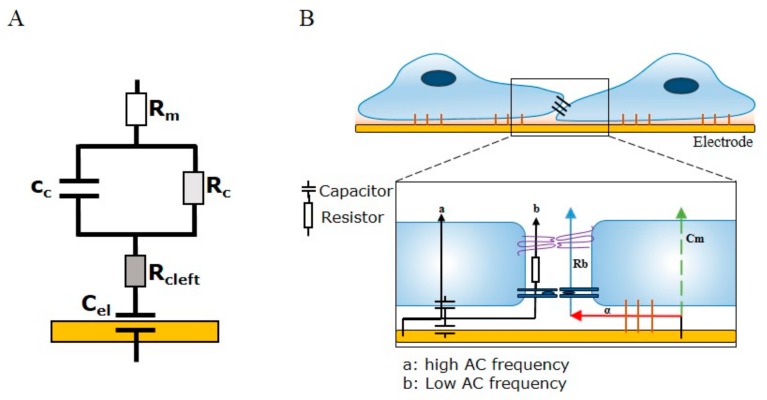
Schematic illustration demonstrating electrical cell-substrate impedance sensing (ECIS) current flow and modelled parameters. (**A**) An equivalent circuit demonstrating how the electrode, cell layer, and respective media compartments are connected within the ECIS model. Modelling for the paracellular barrier (R_b_), basal adhesion (α), and cell membrane capacitance (C_m_) integrate different components of the electrode capacitance (C_el_), the basal cleft resistance (R_cleft_), the cell capacitance and resistance (C_c_ and R_c_, respectively), and the resistance of apical medium (R_m_); (**B**) A schematic of endothelial cells grown on an ECIS electrode. Changes in cell–cell junctions and subcellular adhesion can be measured with ECIS as changes in the current flow at high (>10,000 Hz) and low (4000 Hz) frequencies. A high-frequency current passes through the cell body to couple the capacitive functions of the electrode and cell membrane. Low frequencies take the paracellular route and are resisted by intercellular junctions such as tight junctions and adherens junctions (measured as resistance). Analysis of confluent monolayer properties uses the modelled parameters R_b,_ α, and C_m._ R_b_ is dependent on the collective sum of the intercellular space and the tightness of cell–cell junctions, α is dependent on the cell radius and subcellular adhesion, and C_m_ models changes in the composition of cell membranes.

ECIS involves the growth of cells on top of gold interdigitating electrodes. The application of a weak alternating current (10–10^5^ Hz) through the electrode array provides a means of measuring the ability of cells grown in a monolayer to impede the movement of electrons through and between individual cells. The impedance measurement provides information on two indicators of cell behaviour—resistance (R) and capacitance (C). While capacitance provides measurements related to the overall coverage of the electrode by the cell layer, resistance is indicative of the barrier function of the cells. The principle behind being able to separate impedance (Z) into the two cell-behaviour indicators is dependent on the AC frequency (*f*) passing through the cell layer, as adherent cells are able to alter the flow of current through a monolayer in a frequency-dependent manner. At low frequencies (10^2^–10^4^ Hz), the AC is less able to pass through the cell body due to the capacitive effect of the cell membrane largely resisting current flow (reactance; $X_{c}=\;\frac{1}{2\pi fC}$), forcing the current to flow around the cell bodies and thus through the intercellular space. At low frequencies, therefore, the movement of current between the cells is mostly restricted by the presence of intercellular junctions. Conversely, at high frequencies (>10,000 Hz), the current is able to flow through the cell body due to low reactive capacitance, where the capacitive function of the cell membrane is indicative of the degree of cell coverage over the electrode. Capacitance is useful, as changes in the area (A) of cell membranes and distance (d) of cells above the electrode allow for interpretations of cell attachment, cell spreading, and cell loss changes (as per the formula: capacitance = $\frac{\varepsilon A}{d}$). The power of ECIS is further enhanced by the ability to apply mathematical modelling to derive three parameters that describe three related properties of cells: R_b_ (paracellular barrier), α (basal adhesion), and C_m_ (cell membrane capacitance). Of note, to model the parameter R_b_, four assumptions of cell behaviour need to be met: (I) the electrode needs to be covered by a confluent monolayer; (II) the cells need to have a uniform radius and distance above the electrode; (III) the current needs to be flowing radially within the space between the cells and electrode; and (IV) the current density needs to remain constant [3]. In terms of modelling endothelial barrier function in vitro, of most importance is the parameter R_b_ (Ω cm^2^), as it describes the tightness of the intercellular space, which is highly dependent on cell–cell junctions. The two remaining parameters, α (Ω^0.5^ cm) and C_m_ (μF/cm^2^), are indicative of the current flow below cells or through cells, respectively. The parameter α is useful in describing changes in cell radius and basal adhesion, while C_m_ describes the changes in membrane composition as a function of capacitance. ECIS biosensor technology is the only biosensor technology currently available that can model each of these important cellular parameters. Figure 1 summarises the principles of ECIS and the theory behind barrier integrity measurements and the modelled parameters α, R_b_, and C_m_.

**Figure 2:**
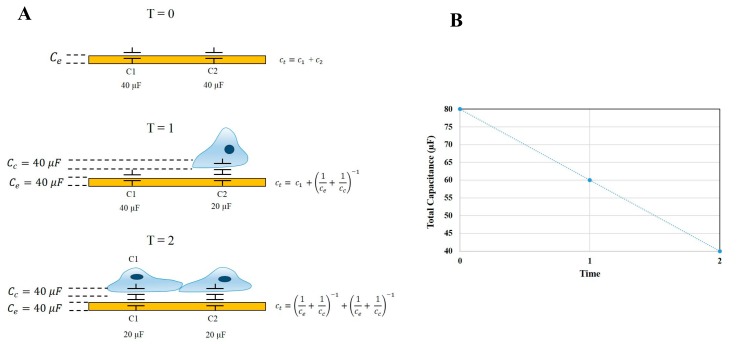
Diagrammatic description of ECIS-Zθ capacitance theory. (**A**) The attachment and cell growth over an ECIS electrode over time. At T = 0 the total capacitance (C_T_) of the system is equal to the sum of the electrode capacitance (C_e_) in parallel. As cells progressively adhere to the surface electrodes, an increasing proportion of C_T_ is represented by the cell-electrode capacitance (C_c_). The cell-electrode capacitance can be interpreted as capacitors connected in series; (**B**) Graphical representation of the change in total capacitance over time, as cells adhere to and cover exposed electrode areas.

An important function of ECIS-Zθ technology is the ability to derive capacitance readings from the measured impedance. Capacitance measurements relate to the ability of the electrode-substrate and cell-substrate interfaces to accumulate charge on their respective surfaces. In a cell-free well, the total capacitance of the system is equivalent to the capacitance of the electrode. However, as cells begin to adhere and cover the electrode arrays, the total capacitance (C_T_) is derived from the combined electrode capacitance (C_e_) and cell capacitance (C_c_). The capacitance of the electrode and cell system can be simplified and represented as being equivalent to two capacitors in series, as per the equation $\frac{1}{C_{T}}=\;\frac{1}{C_{e}}+\;\frac{1}{C_{c}}$. Therefore, as cells progressively cover the gold electrodes, C_T_ is no longer equivalent to C_e_, but instead, a greater proportion of C_T_ is influenced by the two in-series capacitors of the cell-electrode system. Thus, by definition, as C_c_ increases (as a function of the cell area increasing), C_T_ must decrease. This is depicted in Figure 2, where at the point of cell seeding, the capacitance of the system is high and is followed by a steady decrease while cells adhere and spread across the electrodes. Using this theory, any increase in capacitance can be attributed to either an increase in electrode exposure as cells reorganise their junctional space or changes in the physical capacitive properties of the cell membrane, such as changes in composition, area, and thickness.

### 2.3. Conducting ECIS Experiments

ECIS experimental procedure involved pretreating a 96w20idf ECIS plate with 10 mM L-cysteine prior to coating with 1 µg/mL collagen I (Gibco, USA). The endothelial cells were seeded at 20,000 cells per well in 100 µL of media (see figure legend for specific type) and were monitored until a barrier had formed, typically ~48 h post seeding. Upon barrier formation, media was completely removed and replaced with 100 µL of each respective media type being investigated. The endothelial barrier resistance was then monitored for at least 48 h, at which point multifrequency data was collected and modelled using ECIS software (Applied Biophysics, Troy, NY, USA). ECIS measurements were acquired from three independent biological repeats with three replicates per experiment. Shown is one independent experiment with three replicates, which is representative of the three biological repeats. The results were graphed using GraphPad Prism 7.03 software (GraphPad Software Inc., LaJolla, CA, USA) and represented as the average mean ± SD of the three replicates.

### 2.4. Immunocytochemistry

Endothelial cells were seeded onto collagen-I-coated 96-well plates at 20,000 cells per well in either 100 µL Enriched Media or Minimal Media. Immunocytochemistry (ICC) was carried out as companion experiments to ECIS and cells were grown until a barrier had formed as per ECIS recordings. At the point of barrier formation, all media was removed and replaced with each respective test media type. For CD144, Zonula occludens-1 (ZO-1), β-catenin, and α-catenin immunolabelling cells were prefixed in 2% paraformaldehyde (PFA) for 2 min and then fixed in 4% PFA for 10 min. PFA was aspirated and cells were rinsed with PBS prior to permeabilisation with 0.1% Triton-X100 in PBS (PBST) for 10 min. Cells were then washed three times for 10 min in PBS and then blocked with 1% BSA in PBS for 45 min. Following blocking, the cells were washed thrice with PBST and incubated with anti-CD144 (cat# sc-9989, Santa Cruz, Dallas, Texas, USA, 1:200), anti-ZO-1 (cat# 33-9100, ThermoFisher, Waltham, MA, USA, 1:200), anti-β-catenin (cat# 13-8400, ThermoFisher, Waltham, MA, USA, 1:250), or anti-α-catenin (cat# 13-9700, ThermoFisher, Waltham, MA, USA, 1:250) antibodies for 1 h at room temperature. Cells were washed in PBST three times and were incubated with Alexa Fluor 488 conjugated secondary antibody (Goat anti-Mouse, cat# A-11001, Invitrogen, Carlsbad, CA, USA, 1:400) and Hoechst 33342 nuclei stain (R37165, Invitrogen, Carlsbad, CA, 1:10,000) for 1 h at room temperature. Cells were washed for a final three times in PBST and imaged on EVOS FL Auto Imaging System (Invitrogen, Carlsbad, CA, USA) to acquire wide-field images of GFP and DAPI channels. Images were merged using Image J software. Cell-count analysis was carried out using Image J software of Hoechst stained nuclei.

## 3. Statistics

Data are described in the text and are graphically presented as mean ± SEM or mean ± SD for one representative experiment. Each experiment was repeated at least three times and statistical analyses were performed using GraphPad Prism 7.03. ECIS statistical analysis was carried out by determining the effect of treatment on an endothelial monolayer using repeated measures two-way ANOVA with multiple comparisons (main mean effect), followed by a post hoc Tukey Test. Cell number, cell size, and immunolabelling data statistical analysis was carried out using Student’s *t*-test. Graphical representations of *p* values are * *p* ≤ 0.05, ** *p* ≤ 0.01, *** *p* ≤ 0.001, **** *p* ≤ 0.0001.

## 4. Results

### 4.1. Interpretation of ECIS Data

**Figure 3:**
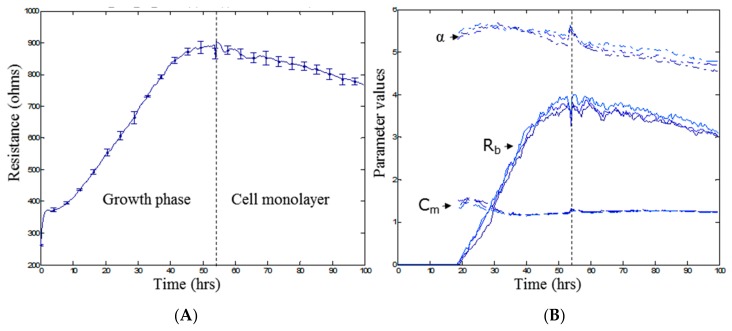
Monitoring parameters R (Ω), R_b_ (Ω cm^2^), α (Ω^0.5^ cm), and C_m_ (µF/cm^2^). (**A**) Time course of resistance magnitude at 4000 Hz for endothelial cells. Influence of the cell growth phase and formation of a cell monolayer on resistance; (**B**) Time course of modelled parameter magnitudes. Illustration of the changes in the three parameters R_b,_ α, and C_m_ as a result of cell growth and monolayer formation as can be seen by an increase in R_b_ overtime. Time point 0 h denotes the time at which cells were seeded at 20,000 cells per well. Data (**A**) show the mean ± SD (n = 3 wells) of one independent experiment representative of three experimental repeats.

Figure 3 shows the typical growth profile of the endothelial cells over the first 100 h following cell seeding into ECIS plates. Figure 3A shows the total resistance (R; ohms) at an AC frequency of 4000 Hz. This measurement reflects the net barrier resistance formed by the endothelial cells, comprising the paracellular barrier (R_b_), basal barrier (α), and the cell membrane (C_m_). Figure 3B shows the multifrequency ECIS data modelled into the R_b_, α, and C_m_ components. The basal adhesion of the endothelial cells to the collagen basement layer forms fast and is maximal by ~20 h. The most important modelled parameter is the R_b_, as it reflects formation of the paracellular junctions between neighbouring endothelial cells. It is evident that R_b_ values do not begin to model until ~20 h after the cells were seeded and reaches a maximum approximately 30 h later. This means that for this particular cell line, a monolayer has formed by ~20 h, but a functional barrier is not present until ~45–50 h after seeding. This barrier remains reasonably stable for the following ~50 h, which reveals the window of experimentation. These data are particularly important for (I) determining that a barrier is present; (II) revealing when the barrier is maximal and can be challenged; and (III) the stability of the barrier as a function of time. The ability of ECIS multifrequency measurements to detect changes in barrier function was validated by the addition of the known barrier modulating factors DMSO and D-Mannitol. Figure S1 highlights the sensitivity of ECIS to temporally monitor a sublethal concentration of DMSO on barrier function and the transient nature of D-Mannitol-induced barrier opening. Understanding the barrier profile of known barrier modulating compounds aids in the interpretation of subsequent barrier modulation by varying culture conditions. 

### 4.2. Influence of Different Culture Media on Barrier Formation of Brain Endothelial Cells Measured Using ECIS Technology

**Figure 4:**
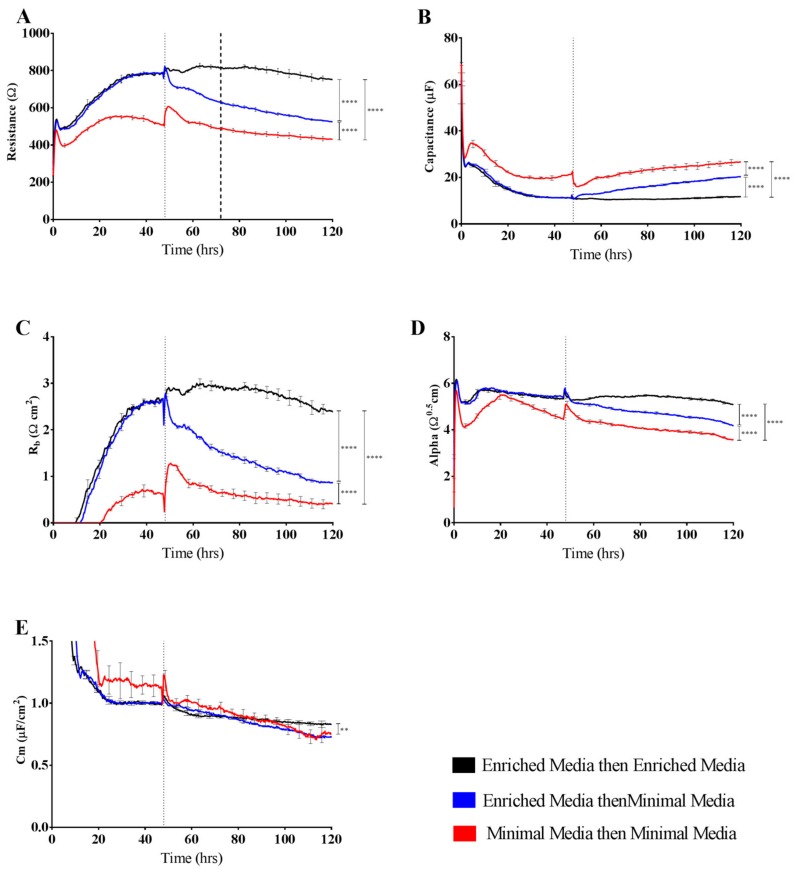
Resistance and modelled parameters R_b_, α, and C_m_ of brain endothelium grown in either Enriched Media or Minimal Media. Endothelial cells were seeded at 20,000 cells per well on a 96w20idf ECIS array. Time 0 h denotes the time cells were seeded. The dotted vertical line indicates 48 h of cell growth in each respective media type, with a subsequent media change carried out at this time. (**A**) Resistance at 4000 Hz trace over 120 h of cell growth; (**B**) Raw capacitance trace over 120 h of cell growth; (**C**) Modelled parameter, R_b_, trace over 120 h of cell growth; (**D**) Modelled parameter, α, trace over 120 h of cell growth; (**E**) Modelled parameter, C_m_, trace over 120 h of cell growth. Data show the mean ± SD (n = 3 wells) of one independent experiment representative of three experimental repeats. Graphical representations of *p* values are * *p* ≤ 0.05, ** *p* ≤ 0.01, *** *p* ≤ 0.001, **** *p* ≤ 0.0001.

Figure 4 shows data from a simple paradigm of growing endothelial cells in different culture media and using ECIS technology to measure the subsequent resistance and barrier formation relative to each media. Resistance measurements taken at 4000 Hz revealed distinct differences in brain endothelial barrier function due to the different media. Medium enriched for growth factors, reputed barrier strengthening compounds, and serum (Enriched Media) resulted in the greatest resistance measurements of ~800 Ω (Figure 4A). Conversely, the removal of the growth factors hEGF and hFGF as well as a reduction in serum concentration in the Minimal Media (red curves) showed a significantly reduced resistance, plateauing around 500–550 Ω. To determine if the changes seen in overall resistance between Enriched Media and Minimal Media were a consequence of changes occurring during the growth phase, cells were grown in Enriched Media until a barrier had formed (~48 h; first dashed line) and then media was removed and replaced with Minimal Media (Figure 4A). An immediate reduction in barrier resistance was observed within 2 h of the change, with the disruption in the endothelial barrier maintained thereafter. Collectively, this suggests that the optimal growth, barrier forming, and sustaining conditions for brain endothelial cells require a combination of growth factors, mitogens, and specific barrier strengthening compounds such as cAMP and hydrocortisone (as present in the Enriched Media).

As impedance measurements allow for the acquisition of both cellular resistance and capacitance readings, interpretation of the changes occurring at the level of capacitance further add to the power of ECIS technology. As with the resistance measurements described above, altering the growth conditions of endothelial cells leads to pronounced changes at the level of cell growth over the electrode arrays, seen as changes in capacitance (Figure 4B). Importantly, it is observed that Minimal Media alters the way in which cells populate the space over electrodes during both growth and barrier forming phases. This can be interpreted as the cells likely existing in a more rounded and less adherent phenotype.

Mathematical modelling of the resistance measurements was conducted to determine the contribution of the paracellular barrier (R_b_), basal attachment (α), and cell membrane (C_m_) to the observed changes. Contributing significantly to the differences in barrier resistance between Enriched Media and Minimal Media are the disparities in paracellular resistance R_b_ (Figure 4C). Endothelial cells cultured in Enriched Media show R_b_ values consistent with what we have observed previously, as per Kho et al. [12]. However, removal of the growth-inducing compounds significantly impairs these interactions, leading to a much weaker paracellular barrier (R_b_). Also notable is the delayed formation of R_b_ for the cells grown in the Minimal Media. Most striking is the rapid loss of paracellular barrier (R_b_) when the cells are switched from Enriched to Minimal Media. The modelled parameter α, which indicates the strength of interaction with the basal substrate, is slightly affected by the Minimal Media but to a much lesser degree than R_b_ (Figure 4D). In contrast, C_m_, which is indicative of temporal alterations in membrane thickness and composition, is not affected by growth in either Enriched or Minimal Media (Figure 4E). As C_m_ measurements are used to determine if changes in capacitance are solely due to changes in electrode coverage or are a function of micro changes in apical membrane structures, the interpretation from the data is that Minimal Media likely affects electrode coverage and not membrane structure. The temporal nature of ECIS and modelling is powerful in revealing these responses.

### 4.3. Systematic Investigation of the Barrier Modulating Effects of Media-Specific Components

**Figure 5:**
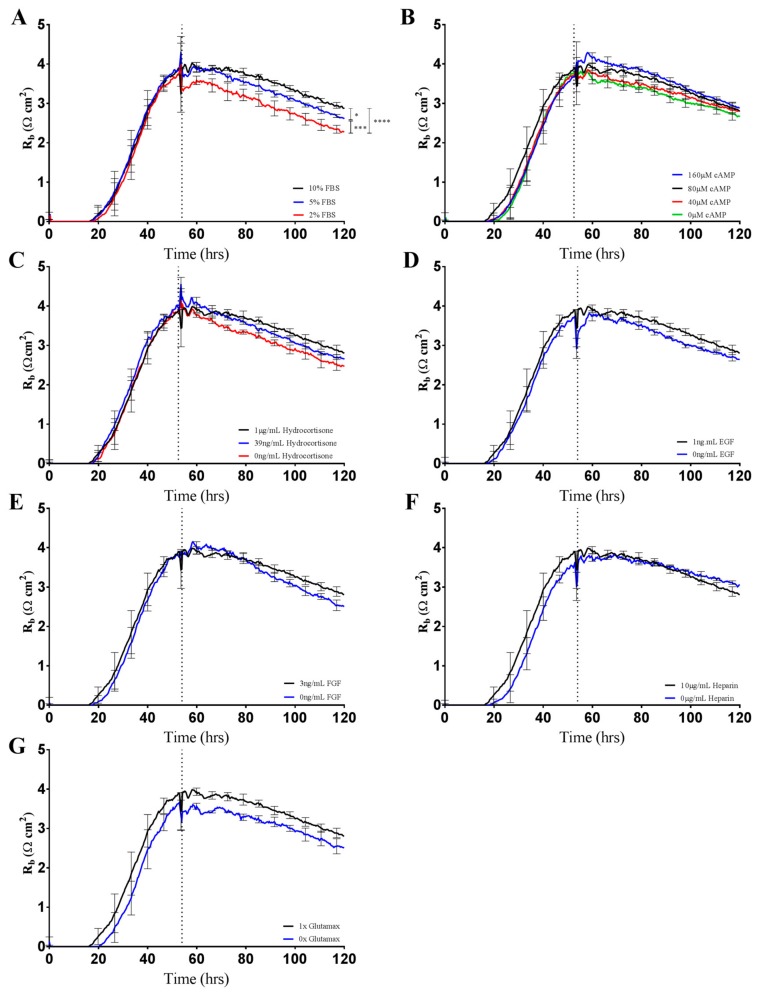
Changes in R_b_ as a consequence of altering growth medium components as shown by brain endothelium. Endothelial cells were seeded at 20,000 cells per well on a 96w20idf ECIS array. Time 0 h denotes the time cells were seeded. The dotted vertical line indicates 48 h of cell growth in each respective media type, with a subsequent media change carried out at this time. (**A**) Brain endothelium grown in Enriched Media containing either 10%, 5%, or 2% FBS; (**B**) Brain endothelium grown in Enriched Media containing either 160 µM, 80 µM, 40 µM, or 0 µM cAMP; (**C**) Brain endothelium grown in Enriched Media containing either 1 µg/mL, 39 ng/mL, or 0 ng/mL hydrocortisone; (**D**) Brain endothelium grown in Enriched Media containing either 1 ng/mL or 0 ng/mL EGF; (**E**) Brain endothelium grown in Enriched Media containing either 3 ng/mL or 0 ng/mL FGF; (**F**) Brain endothelium grown in Enriched Media containing either 10 µg/mL or 0 µg/mL heparin; (**G**) Brain endothelium grown in Enriched Media containing either 1× or no Glutamax. Data show the mean ± SD (n = 3 wells) of one independent experiment representative of three experimental repeats. Graphical representations of *p* values are * *p* ≤ 0.05, ** *p* ≤ 0.01, *** *p* ≤ 0.001, **** *p* ≤ 0.0001.

The contribution of each media supplement to the overall resistance of Enriched-Media-cultured endothelial cells was revealed by ECIS measurements at 4000 Hz (Figure 5). At the formation of a strong endothelial monolayer, normal Enriched Media was removed and replaced with a variation of the media type in order to separate the effects of each medium component. The only noticeable change in paracellular resistance (as per R_b_) was a concentration-dependent response to FBS, with a 20% decrease in resistance from 10% to 2% FBS (Figure 5A). However, it is important to note that the difference in barrier resistance between 10% FBS and 2% FBS does not solely account for the decrease in resistance seen when media is changed from Enriched Media to Minimal Media (Figure 4A). While significant decreases in paracellular barrier resistance were observed when endothelial cells were grown in decreasing concentrations of FBS, other critical Enriched Media components failed to alter barrier integrity in isolation (Figure 5B–G). The findings are suggestive of synergistic, opposed to additive, effects by the supplemented media components to overall endothelial barrier resistance. The establishment of a strong in vitro brain endothelial barrier is, therefore, likely to be regulated by the milieu of media components in a complex and interactive manner.

### 4.4. Immunocytochemistry Validates ECIS Findings

To validate the findings of altered cell–cell interactions indicated by the ECIS measurements, classical immunocytochemistry was performed in conjunction with ECIS recordings. We hypothesised that the substantial differences in R_b_ observed between the Enriched Media and Minimal Media (Figure 4C) would be reflected in expression and localisation of the key junctional proteins CD144, ZO-1, and the accessory catenins.

**Figure 6:**
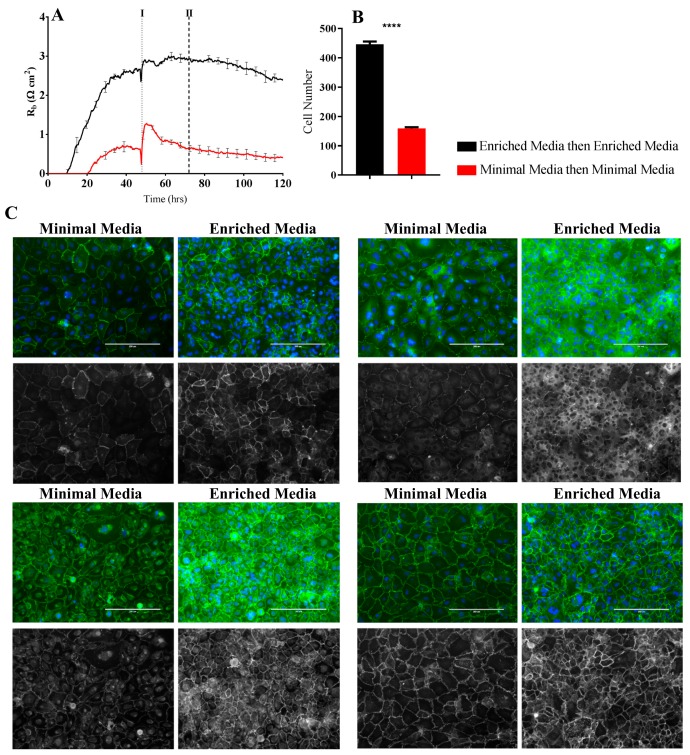
Immunocytochemistry of brain endothelial junctional space following growth in either Enriched Media or Minimal Media. Cells were seeded at time 0 h at 20,000 cells per 0.3 cm^2^. (**A**) ECIS resistance and R_b_ measurements over 120 h. Cells were grown in either Enriched Media or Minimal Media from T = 0 h, with media changed at T = 48 h (I). Data shown is the mean ± SD (n = 3 wells) of one independent experiment representative of three experimental repeats; (**B**) Cell number count of Hoechst stained nuclei at time point II, obtained through Image J Software analysis. Data shown is the mean ± SEM (n = 18 wells) of 1 independent experiment representative of 3 experimental repeats; (**C**) The junctional space following growth in either Enriched Media or Minimal Media 72 h post seeding. The time point of fixation corresponds to the second vertical dotted line (II) shown on the ECIS traces. Representative GFP/DAPI and GFP monochrome images for the junctional proteins CD144, ZO-1, β-catenin, and α-catenin are shown. Each panel shows cells grown in Minimal Media on the left and cells grown in Enriched Media on the right. The corresponding Alexa Fluor 488 (GFP) monochrome image is shown below each merged image. Green—junctional protein, blue—nuclei. Scale bar is 200 µm. Immunocytochemistry data show one representative image from one independent experiment, which is representative of three experimental repeats. Graphical representations of *p* values are * *p* ≤ 0.05, ** *p* ≤ 0.01, *** *p* ≤ 0.001, **** *p* ≤ 0.0001.

**Figure 7:**
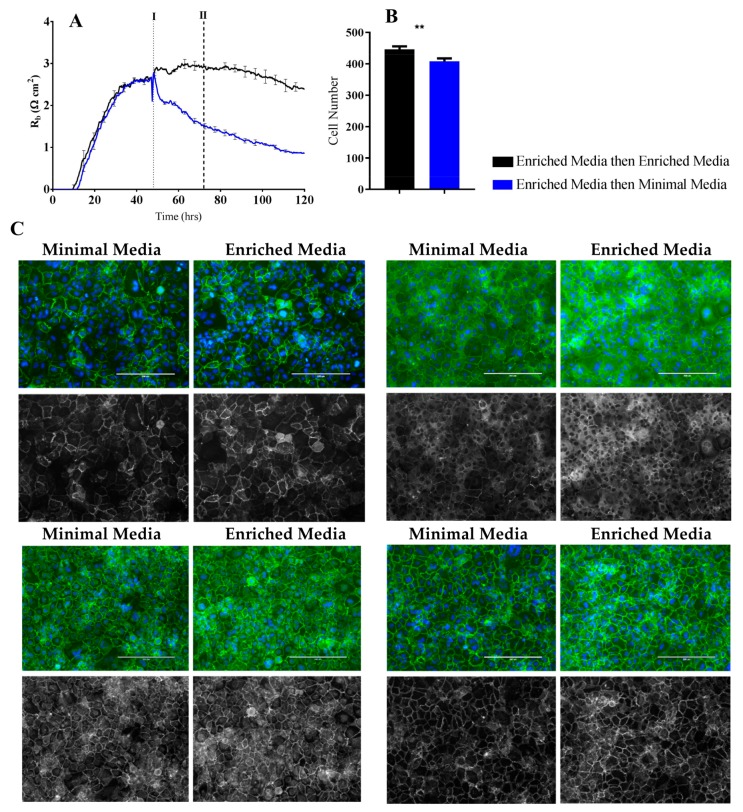
Immunocytochemistry of brain endothelial junctional space following growth in either Enriched Media or Minimal Media. Cells were seeded at time 0 h at 20,000 cells per 0.3 cm^2^. (**A**) ECIS resistance and R_b_ measurements over 120 h. Cells were grown in either Enriched Media or Minimal Media from T = 0 h, with media changed at T = 48 h (I). Data shown is the mean ± S.D. (n = 3 wells) of 1 independent experiment representative of 3 experimental repeats; (**B**) Cell number count of Hoechst stained nuclei at time point II, obtained through Image J Software analysis. Data shown is the mean ± SEM (n = 18 wells) of 1 independent experiment representative of 3 experimental repeats; (**C**) The junctional space following growth in either Enriched Media or Minimal Media 72 h post seeding. The time point of fixation corresponds to the second vertical dotted line (II) shown on the ECIS traces. Representative GFP/DAPI and GFP monochrome images for the junctional proteins CD144, ZO-1, β-catenin and α-catenin are shown. Each panel shows cells grown in Minimal Media on the left and cells grown in Enriched Media on the right. The corresponding Alexa Fluor 488 (GFP) monochrome image is shown below each merged image. Green—junctional protein, blue—nuclei. Scale bar is 200 µm. Immunocytochemistry data shows 1 representative image from 1 independent experiment, which is representative of 3 experimental repeats. Graphical representations of *p* values are * *p* ≤ 0.05, ** *p* ≤ 0.01, *** *p* ≤ 0.001, **** *p* ≤ 0.0001.

As hypothesised, the larger the differences in paracellular resistance as shown by ECIS R_b_, the more apparent the changes in the junctional space appear as per immunolabelling for key tight junction and adherens junction molecules (Figure 6 and Figure 7). In particular, regardless of the time-point assessed by ICC, the cells grown in the Enriched Media consistently show higher expression of the junctional molecules, in conjunction with more homogenous junctional structures (Figure 6 and Figure 7C, Figure S2 data). The finding corresponds with ECIS R_b_ measurements, whereby a higher endothelial paracellular resistance translates to an increase in junctional proteins within the intercellular space (Figure 6 and Figure 7A). Of note is also the evidence of increased levels of cytoplasmic localisation of tight junction (ZO-1) and adherens junction (α-, β-catenin) regulatory molecules when cells are grown in the Enriched Media (Figure 6 and Figure 7C).

An additional observation is the apparent decrease in cell size during the growth phase when cells are grown in Enriched Media (Figure 6 and Figure 7C, Figure S3 data). The decrease in cell size correlates to an absolute increase in the monolayer cell number within the same area, which is expected due to the presence of growth factors and mitogens within Enriched Media (Figure 6 and Figure 7B). Contrary to ECIS theory, the increase in absolute cell number does not correlate to a decrease in R_b_, but instead an increase. Typically, an increase in cell number would create an increase in paracellular volume which subsequently allows greater current to flow through the junctional space, seen as reduced paracellular endothelial resistance. However, when cells are grown in Enriched Media, there is a significant increase in R_b_ in conjunction with greater cell number. Therefore, Enriched Media is likely resulting in strengthening of intercellular adhesions to override the increased paracellular volume phenomenon. Evidently, mechanisms at the level of the cell–cell junctions are promoting the greater endothelial barrier strength, and this is consistent with our junctional staining data.

## 5. Discussion

Endothelial cells line all of the blood and lymphatic structures throughout the body. They are phenotypically highly variable across tissues where they form selective barriers, the strength of which also varies across tissues [13]. A major area of endothelial biology is understanding how these barriers are formed and regulated in both health and disease [14]. In this paper, we highlight the power of ECIS biosensor technology to measure and model multiple aspects of brain endothelial barrier formation in a real-time autonomous manner. These aspects include determining (I) when the barrier has formed; (II) when the barrier is at its strongest; (III) temporal stability of the barrier; and (IV) as demonstrated here, the culture conditions required to produce a strong stable barrier. Importantly, we highlight the sensitivity of the technology to detect real-time changes occurring within the paracellular space (defined as R_b_), which we can attribute to molecular changes in the expression of specific junctional proteins. 

ECIS technology is an impedance biosensor capable of applying a very low alternating current at multiple frequencies (from 10 to 10^5^ Hz) [3]. The two direct measurements that are produced are resistance and capacitance. In terms of endothelial cells, impedance measured across multiple frequencies can be mathematically modelled using the ECIS software to derive several important parameters which collectively relate to the overall barrier formation of the cells. These are R_b_ (resistance of the paracellular space), α (resistance due to basal adhesion), and C_m_ (capacitance of the cell membrane). ECIS is the only impedance biosensor to use multifrequency AC. This is a major distinguishing factor in comparison to xCELLigence technology from ACEA, which generates the data from a single AC frequency. Therefore, although xCELLigence can measure these parameters collectively (total resistance), it cannot model which aspect of barrier function changes [7]. 

Understanding which components of the barrier are contributing to the barrier strength and the resistance measured by ECIS is very important. Consider the scenario where endothelial cells have very strong basal adhesion but weak paracellular adhesion, which would be expected of permeable lymphatic endothelial cells [15]. This will produce a high resistance, primarily derived from the basal resistance. However, endothelial cells of the blood brain barrier (BBB) have a strong paracellular barrier governed by junctional proteins, as well as strong basal adhesion. Therefore, for in vitro studies, it is highly pertinent to know: (I) when the endothelial barrier has formed, which is not simply when a monolayer is visible; (II) the stability and duration of that the barrier; and (III) the strength of both the paracellular and basal barriers.

It is predictable that the formation and maintenance of a strong endothelial barrier in vitro will be dependent on the optimal use of growth factors and supplements in the cell-culture media and that this will vary depending on cell type and source. We hypothesised that our immortalised brain endothelial cells cultured in media enriched with various growth factors and 10% FBS (Enriched Media) would exhibit a stronger and more stable barrier than that produced from a less enriched media (Minimal Media). This was indeed observed where Enriched Media achieved an endothelial resistance of ~800 Ω, whereas the Minimal Media only achieved a maximal resistance of ~500 Ω (Figure 4A). It was unclear as to which components of the barrier were influenced to a greater extent by the Enriched Media or which factors within the media caused the greater effect. This is where the power of ECIS really excelled, as it clearly demonstrates that the paracellular barrier (R_b_) is dramatically weaker in the cells grown in the Minimal Media. The endothelial cells grown in the Enriched Media also begin to form their paracellular barrier faster and achieve a stronger barrier, which is maintained for at least 50–60 h (stronger for longer). The basal adhesion (α) is weaker for the cells grown in the Minimal Media, but this effect is far less dramatic than that of R_b_. The real-time power of ECIS is highlighted in Figure 4, where the brain endothelial cells cultured in the Enriched Media are switched to the Minimal Media. In this scenario, there is an immediate and continuous loss of paracellular barrier strength and basal adhesion. This not only highlights the importance of real-time measurements for observing immediate or acute changes in barrier function but reveals that the maintenance of the stronger barrier is a highly active process continually requiring the nutrients present in the Enriched Media [16,17]. 

Typically, optimisation of BBB endothelial in vitro models has relied on transendothelial electrical resistance (TEER) as an indication of increased paracellular resistance [10,11,18,19]. In particular, epithelial voltohmmeter (EVOM) has been extensively employed as a means of quantitatively describing barrier integrity based on ohmic resistance. The extensive limitations of EVOM are reviewed elsewhere [20], however it is worth noting that EVOM TEER measurements provide measurements only based on the overall resistive properties of an endothelial cell monolayer grown on porous membranes. Consequently, EVOM recordings do not allow for the separation of paracellular and basal resistance, thus limiting the interpretation of subsequent TEER measurements [20,21]. ECIS, therefore, as a means of establishing the conditions required to generate a strong brain endothelial barrier, provides a more in-depth and relevant analysis of cell barrier properties (described by [2]).

ECIS technology further allowed us to determine the contribution of each Enriched Media additive to the increased barrier resistance by brain endothelial cells (Figure 5). Unexpectedly, serum concentration appeared to be the only additive that significantly altered endothelial barrier strength (Figure 5A). Serum-dependent barrier function is well established throughout the literature [10,11,18,22,23]. However, serum addition is frequently reported to negatively affect blood brain barrier integrity, with high concentrations of serum and the presence of growth factors within the culture medium reportedly capable of inhibiting the formation of brain endothelial tight junctions [24]. In a unique study, Nitz et al. demonstrated that the disruption of tight junctions by serum was highly side specific, meaning that apically applied serum did not decrease barrier function to the same extent as basal administration [24]. Physiologically, apical membranes of brain endothelium are exposed to serum at much higher concentrations compared to the basal compartments. The side-specific nature of serum-mediated junctional disruption likely reflects membrane-specific receptor–serum interactions at the basal surface that mediate vascular leakiness.

Measuring endothelial barrier resistance with ECIS allows impedance measurements to be acquired when cells are grown on a flat, nonporous surface coated with appropriate extracellular matrix proteins. Therefore, unlike typical EVOM measurements, where cells are required to be grown on porous filters, ECIS ensures serum is only exposed to the apical surface of brain endothelium, thus eliminating any potential for basal–serum interactions that possibly promote loss of barrier integrity. A recent systematic review of immortalised brain endothelial cell lines showed that out of 49 studies investigating endothelial barrier resistance, 48 used transwell-based systems [25]. Most of these studies had serum present on both sides of the polarised endothelial cells. High serum levels on the basolateral aspect of the endothelium is not physiological and potentially may introduce a barrier artefact in some studies. There is also a need for a better understanding of side-specific serum effects on brain endothelial cells. ECIS technology using solid-state plates negates the potential limitations of transwell-based resistance measuring systems, making it an ideal tool for these studies. The 96-well ECIS plates also offer the capacity to assess a wide range of culture and growth conditions simultaneously, ideal for optimisation. 

The ECIS modelling clearly shows that the paracellular barrier is stronger for longer in the Enriched Media. Such a sizable difference should be reflected at the molecular level in the expression of key junctional proteins that govern the strength of the paracellular space [26]. Others have recommended that changes occurring in R_b_ measurements should be critically assessed by immunocytochemical staining of the endothelial cell monolayer [3] to validate and confirm the modelled parameters. We therefore assessed the expression and location of key junctional proteins (CD144, ZO-1, β-catenin, and α-catenin) which are known to positively contribute to paracellular barrier strength (Figure 6 and Figure 7). There was a striking and obvious difference in the location and expression level of each of these key proteins after 72 h of culture in the respective media. Overall, the expression is higher in the Enriched Media, with pronounced junctional staining evident for CD144, α-catenin, and β-catenin. There is also a greater number of cells present, which means there is a greater volume of paracellular space present under these conditions (Figure 6B). This, in theory, provides more path for current flow between the cells. Therefore, in order for the R_b_ to be higher in the Enriched conditions, it means that the barrier must be tighter. In the scenario, where the media was switched from Enriched to Minimal Media, there is a substantial loss of R_b_. There is also a concordant reduction in the expression of CD144, ZO-1, α-catenin, and β-catenin. The most obvious differences of these are for CD144 and ZO-1. Although these are subtler than the differences where the cells are grown entirely in the Minimal Media, the changes are consistent with the modelled differences in R_b_. 

We conclude that ECIS provides a powerful multiparametric method of assessing endothelial barrier function, and that this solid-substrate impedance sensing technology has multiple advantages over other technologies. As our study requires an understanding of the changes occurring in both the paracellular space and contribution from the basal adhesion, other technologies such as EVOM or xCELLigence RTCA would not have sufficed. The utility of ECIS has provided us with a powerful means of assessing multiple aspects of endothelial barrier formation, maintenance, stability, and the optimal conditions required for the generation of a strong barrier (stronger for longer). Importantly, the investigation highlighted the importance of multifrequency impedance sensing in monitoring endothelial barrier function, with emphasis placed on the biological context of ECIS technology.

## Supplementary Materials

The following are available online at http://www.mdpi.com/2079-6374/8/3/64/s1, Figure S1: DMSO- and D-Mannitol-induced endothelial permeability. Endothelial cells were seeded at 20,000 cells per well on a 96w20idf ECIS array. Time 0 h denotes the time cells were seeded. The dotted vertical line indicates 48 h of cell growth in each respective media type, with a subsequent media change carried out at this time. (**A**) Resistance at 4000 Hz of DMSO- (0.5%) and D-Mannitol- (100 mM) treated endothelial cells. (**B**) R_b_ of DMSO- (0.5%) and D-Mannitol- (100 mM) treated endothelial cells. (**C**) Alpha of DMSO- (0.5%) and D-Mannitol- (100 mM) treated endothelial cells. Data show the mean ± SD (n = 3 wells) of one independent experiment representative of three experimental repeats, Figure S2: Quantitative analysis of immunolabelled endothelial cells cultured in either Enriched Media or Minimal Media. Cells were cultured in the first media condition for 48 h and then the second for a further 48 h and immunolabelled for junctional molecules. CD144, ZO-1, α-catenin, and β-catenin 488 Alexa Fluor GFP image fluorescent intensity measurements acquired through ImageJ software analysis. (**A**) CD144 mean gray values of endothelial cells grown in altered media conditions. (**B**) ZO-1 mean gray values of endothelial cells grown in altered media conditions. (**C**) α-catenin mean gray values of endothelial cells grown in altered media conditions. (**D**) β-catenin mean gray values of endothelial cells grown in altered media conditions. Data show the mean ± SD (n = 3 independent experiments), Figure S3: Quantitative cell size analysis of immunolabelled endothelial cells cultured in either Enriched Media or Minimal Media. ImageJ software analysis of endothelial cell size. Cells were cultured in the first media condition for 48 h and then the second for a further 48 h. Junction specific immunolabelling with anti-α-catenin antibodies allowed for demarcation of cell membranes. Images were acquired at 20× magnification and ImageJ analysis was calibrated to a 200 µm scale bar. (**A**) Cell area of endothelial cells grown in altered media conditions. (**B**) Cell circumference of endothelial cells grown in altered media conditions. Data show the mean ± SD (n = 3 independent experiments).
