# Supplementary material for: The Importance of Multifrequency Impedance Sensing of Endothelial Barrier Formation Using ECIS Technology for the Generation of a Strong and Durable Paracellular Barrier
Source: Biosensors (Basel). 2018 Jul 4;8(3):64. doi: 10.3390/bios8030064 (PMC6163417; doi:10.3390/bios8030064)
Supplement: Supplementary file 1 [file biosensors-08-00064-s001.pdf]

biosensors

Supplementary Figures:

The Importance of Multifrequency Impedance Sensing of Endothelial Barrier Formation Using ECIS Technology for the Generation of a Strong and Durable Paracellular Barrier

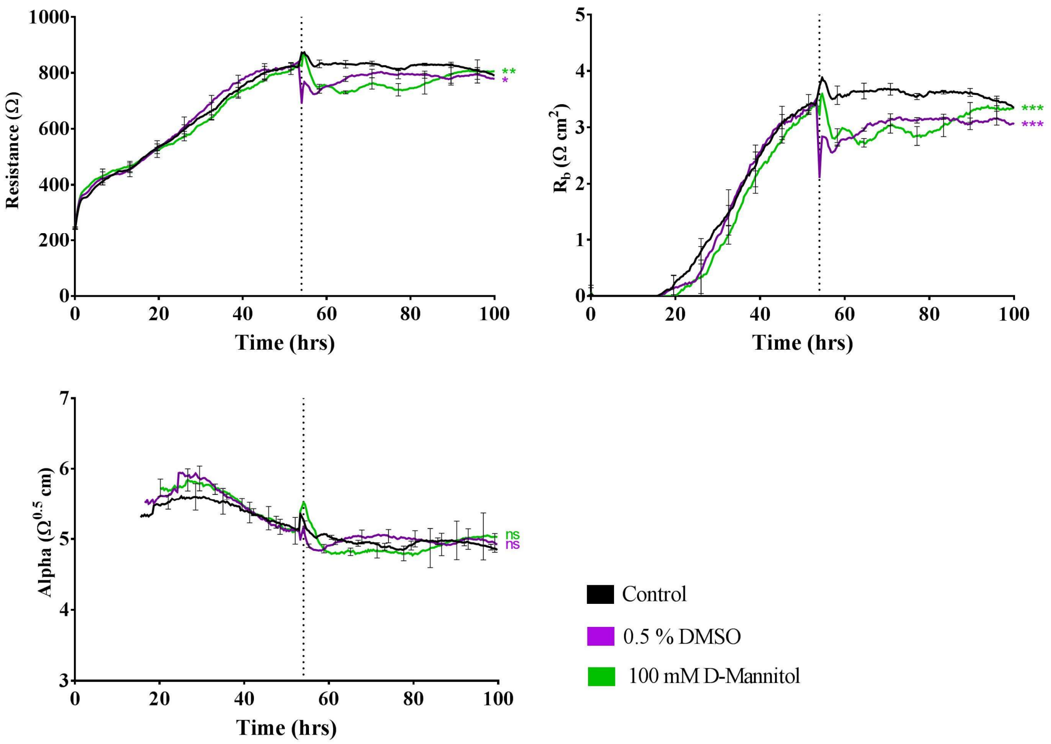

Figure S1. DMSO- and D-Mannitol-induced endothelial permeability. Endothelial cells were seeded at 20,000 cells per well on a 96w20idf ECIS array. Time 0 h denotes the time cells were seeded. The dotted vertical line indicates 48 h of cell growth in each respective media type, with a subsequent media change carried out at this time. (**A**) Resistance at 4000 Hz of DMSO- (0.5%) and D-Mannitol- (100 mM) treated endothelial cells. (**B**) R_b of DMSO- (0.5%) and D-Mannitol- (100 mM) treated endothelial cells. (**C**) Alpha of DMSO- (0.5%) and D-Mannitol- (100 mM) treated endothelial cells. Data show the mean ± SD (n = 3 wells) of one independent experiment representative of three experimental repeats.

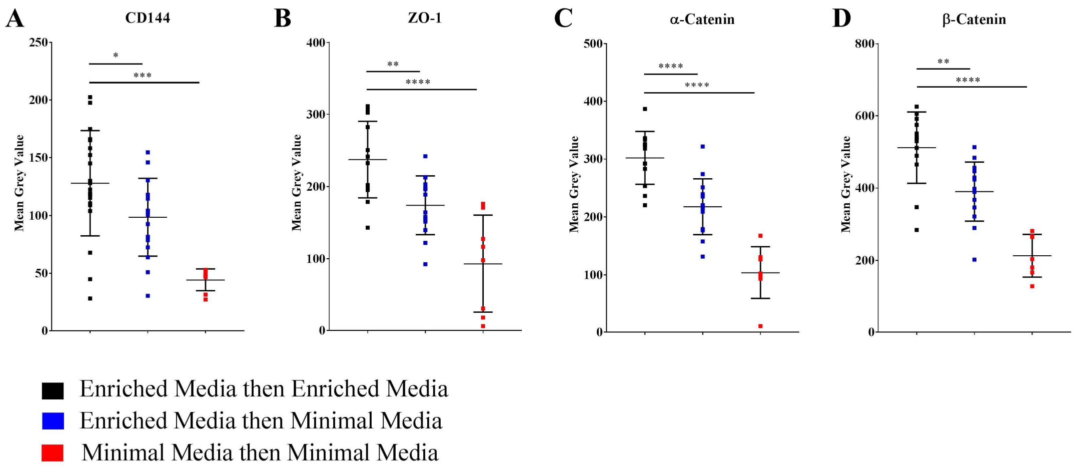

Figure S2. Quantitative analysis of immunolabelled endothelial cells cultured in either Enriched Media or Minimal Media. Cells were cultured in the first media condition for 48 h and then the second for a further 48 h and immunolabelled for junctional molecules. CD144, ZO-1, α-catenin, and β-catenin 488 Alexa Fluor GFP image fluorescent intensity measurements acquired through ImageJ software analysis. (**A**) CD144 mean gray values of endothelial cells grown in altered media conditions. (**B**) ZO-1 mean gray values of endothelial cells grown in altered media conditions. (**C**) α-catenin mean gray values of endothelial cells grown in altered media conditions. (**D**) β-catenin mean gray values of endothelial cells grown in altered media conditions. Data show the mean ± SD (n = 3 independent experiments).

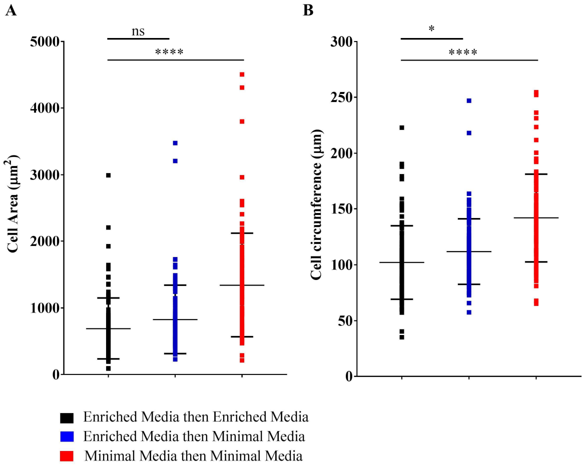

Figure S3. Quantitative cell size analysis of immunolabelled endothelial cells cultured in either Enriched Media or Minimal Media. ImageJ software analysis of endothelial cell size. Cells were cultured in the first media condition for 48 h and then the second for a further 48 h. Junction specific immunolabelling with anti-α-catenin antibodies allowed for demarcation of cell membranes. Images were acquired at 20x magnification and ImageJ analysis was calibrated to a 200 μm scale bar. (**A**) Cell area of endothelial cells grown in altered media conditions. (**B**) Cell circumference of endothelial cells grown in altered media conditions. Data show the mean ± SD (n = 3 independent experiments).
